# Measuring the quality of MDT working: an observational approach

**DOI:** 10.1186/1471-2407-12-202

**Published:** 2012-05-29

**Authors:** Cath Taylor, Louise Atkins, Alison Richardson, Ruth Tarrant, Amanda-Jane Ramirez

**Affiliations:** 1 Florence Nightingale School of Nursing & Midwifery, King’s College London, SE1 8WA, London, England; 2 Centre for Outcomes Research and Effectiveness, University College London, WC1E 7HB, London, England; 3 Faculty of Health Sciences, University of Southampton and Southampton University Hospital Trusts, SO16 6YD, Southampton, England; 4 Department of Clinical Oncology, Southampton University Hospitals NHS Trust, SO16 6YD, Southampton, England; 5 Promoting Early Presentation Group, King’s College London, St Thomas’ Hospital, SE1 7EH, London, England

**Keywords:** Cancer, Multidisciplinary Communication, Interprofessional relations, Observation, Quality Indicators Health Care, Decision-making, Leadership, Health resources

## Abstract

**Background:**

Cancer multidisciplinary teams (MDTs) are established in many countries but little is known about how well they function. A core activity is regular MDT meetings (MDMs) where treatment recommendations are agreed. A mixed methods descriptive study was conducted to develop and test quality criteria for observational assessment of MDM performance calibrated against consensus from over 2000 MDT members about the “characteristics of an effective MDT”.

**Methods:**

Eighteen of the 86 ‘Characteristics of Effective MDTs’ were considered relevant and feasible to observe. They collated to 15 aspects of MDT working covering four domains: the team (e.g. attendance, chairing, teamworking); infrastructure for meetings (venue, equipment); meeting organisation and logistics; and patient-centred clinical decision-making (patient-centredness, clarity of recommendations). Criteria for rating each characteristic from ‘very poor’ to ‘very good’ were derived from literature review, observing MDMs and expert input. Criteria were applied to 10 bowel cancer MDTs to assess acceptability and measure variation between and within teams. Feasibility and inter-rater reliability was assessed by comparing three observers.

**Results:**

Observational assessment was acceptable to teams and feasible to implement. Total scores from 29 to 50 (out of 58) highlighted wide diversity in quality between teams. Eight teams were rated either ‘very good/good’ or ‘very poor/poor’ for at least three domains demonstrating some internal consistency. ‘Very good’ ratings were most likely for attendance and administrative preparation, and least likely for patient-centredness of decision-making and prioritisation of complex cases. All except two characteristics had intra-class correlations of ≥0.50.

**Conclusions:**

This observational tool (MDT-OARS) may contribute to the assessment of MDT performance. Further testing to confirm validity and reliability is required.

## Background

Cancer multidisciplinary teams (MDTs) are well established in the UK and other countries as a core mechanism for improving patient outcomes. A core activity is regular MDT meetings (MDMs), held weekly for most tumour types, which brings together all relevant health professionals to discuss and agree patient treatment plans.

The benefits of MDTs in relation to improved adherence to evidence-based guidelines, better treatment decisions, and association with better clinical outcomes including survival have been well documented [1,2]. Nevertheless MDTs are a very expensive resource and we know little about how well they individually function. The UK national annual cancer peer review programme provides a mandatory mechanism for assessment of MDTs. MDTs have to show their compliance with standards that are derived from tumour-specific improving outcomes guidance (which is in turn based on evidence and/or clinical consensus). Data from the peer-review programme demonstrates wide variation between teams in adherence to standards [3,4]. The standards encompass structural features of MDTs such as team composition (having the required expertise) and having protocols for referral and treatment but omit other aspects of MDT functioning such as the quality of leadership and chairing; teamwork (e.g. inclusiveness and mutual respect); and the clinical decision-making process.

Evidence is accruing regarding the association between process and outcomes in cancer MDTs [2]. The quality of diagnostic and treatment decisions has been related to MDT discussion in a range of tumour types [5-7]. The quality of MDT recommendations relies upon considera-tion of all relevant information. Failure to consider patient-based information has been shown to be a major reason for non-implementation of recommendations, either due to being unacceptable to patients or clinically inappropriate [8-11]. Moreover, non-implementation of MDT meeting recommendations can have both clinical and financial consequences if further discussion is required and treatment is delayed. This is likely to require inclusive discussions in MDT meetings but research has shown that cancer MDT meetings may prioritise the medical model and place less value on the contributions of non-medical members of the team [12,13].

Robust assessment of the complex behaviours and activi-ties in MDMs is likely to require mixed methods including independent observation [14,15]. Observational measures have been developed to assess teamwork in other healthcare teams, such as surgical teams [16,17]. To apply such methods to MDTs first requires agreement about the optimal MDT characteristics. Determining causal relationships between teamworking and outcome is riddled with methodological challenges, which may partly account for the lack of empirical research regarding predictors of effectiveness [1]. Nevertheless, consensus of opinion in a recent UK national survey completed by over 2000 MDT members [18] resulted in recommendations for MDT working: ‘The characteristics of an effective MDT’ [19]. Eighty-six ‘characteristics of effectiveness’ are organised within five domains: The Team; Infrastructure for meetings; Meeting organisation and logistics; Clinical decision-making; and Clinical governance. These characteristics provide a framework against which to develop objective criteria for assessing the quality of teamworking in MDMs.

### Aims

· To develop quality criteria for assessment of characteristics observable in MDMs

· To conduct preliminary tests of their acceptability with 10 bowel cancer teams, to include describing:

∘ the variation *within* and *between* teams

∘ the characteristics most/least likely to receive high quality ratings

· To test inter-rater reliability and feasibility

## Methods

### Development of MDT-OARS (Observational Assessment Rating Scale)

Each of the 86 ‘characteristics of effective MDTs’ [19] was considered in turn for its relevance and feasibility for independent observational measurement in MDT meetings. Additional consideration was given to whether the characteristics were already measured in national peer review assessments, and priority was given to including characteristics that did not overlap with peer review. In total 18 of the 86 characteristics were felt to be observable. Due to overlap between characteristics these aggregated into 15 aspects of teamworking (Table 1).

**Table 1:** MDT-OARS quality criteria and shortened manual for rating each of 15 observable characteristics of effective teamworking in MDT meetings

**Characteristic of effective MDT working**	**Quality Criteria**	**Quality of team working (score in brackets)**				
		**Very Poor**	**Poor**	**Good**	**Very Good**	
**The Team**						
Attendance	Presence of relevant core team members at the meeting	at least one core team member (and their deputy) is not present for the whole meeting	at least one core team member (and deputy) is absent for most of the meeting (≥3 cases)	at least one core team member (and deputy) is absent for part of the meeting (≤ 2 cases)	all core team members (or deputy) present for whole meeting	
		(1)	(2)	(3)	(4)	
Leadership: chairing of meeting	· Keeps meeting to agenda (i.e. moves onto next case)	Satisfies none of criteria	Only satisfies 1–2 of criteria	Satisfies 3 of criteria	Evidence of all of the criteria	
	· Encourages overall participation					
	· Encourages focussed discussion					
	· Articulates recommendation	(1)	(2)	(3)	(4)	
Teamworking & culture						
a) Inclusion of relevant team members	· All relevant core members are actively and appropriately involved	Satisfies 1/none of criteria	Satisfies 2–4 of criteria	Satisfies “*all relevant core members are actively and appropriately involved*” and at least 3 other criteria	Satisfies all of the criteria	
	· Meeting not dominated by 1-2 people					
	· Input/questions volunteered and encouraged					
	· Contributions facilitate decision-making and/or inform discussion					
	· Consensus of decision-making	(1)	(2)	(3)	(4)	
b) Team Sociability	· Evidence of humour	Satisfies none of the criteria	Satisfies 1 of criteria	Satisfies 2–3 of criteria	Satisfies all of criteria	
	· Team appear relaxed with each other					
	· Warm and supportive team environment					
	· Friendly and cooperative communicative style	(1)	(2)	(3)	(4)	
c) Mutual respect	· Focussed attention	Only satisfies 1 or none of	Satisfies 2–3 of criteria	Evidence of respect,	Strong evidence of	
	· Respect for speaker	criteria		evidence of at least 4	respect in all/almost all	
	· No concurrent discussions			criteria	cases	
	· Asking and valuing relevant contributions					
	· General sense of politeness/courtesy (inc mobile phone etiquette)	(1)	(2)	(3)	(4)	
d) tension and conflict		*Not rated on the same scale – see bottom of table*				
Personal development & training	Observable communication of research evidence and/or instances of learning	No observable communication of research evidence or instances of learning	Minimal communication of research evidence or instances of learning		Structured presentation of research evidence and/or learning through formal discussion (e.g. of audit findings	
		(1)	(2)		(3)	
**Infrastructure for meetings**						
Meeting venue	· Room size appropriate for number of team members	Satisfies only 1 or none of criteria	Satisfies 2 of the criteria	Satisfies 3 of the criteria	Satisfies all of the criteria	
	· Layout of chairs enables accessible viewing of diagnostics					
	· Layout of room allows accessible viewing of other team members					
	· All members seated on a chair					
	· Suitable venue in terms of location, temperature, lighting etc	(1)	(2)	(3)	(4)	
Technology & equipment	Availability of diagnostic equipment to view and share images and pathology with the team.	No radiology imaging facilities	Light box available with hard copy film	Current images available digitally with facilities for projecting/viewing images	Current images available digitally with facilities for projecting/viewing images and capability of accessing retrospective images (e.g. use of PACS)	
	Availability of multiple screens scores extra 1 point. Score out of possible 9 is then standardised onto 1-4 scale to give overall rating.					
		(1)	(2)	(3)	(4)	
		No histopathology facilities	Microscope	Microscope with facilities for projecting/viewing specimen/biopsy	Microscope with facilities for projecting and viewing specimen/biopsy and accessing retrospective data	
		(1)	(2)	(3)	(4)	
**Meeting organisation and logistics**						
Preparation prior to meetings:						
a) agenda	Availability and content of agenda	No available agenda	Agenda, but limited info		Comprehensive agenda	
		(1)	(2)		(3)	
b) prioritisation of complex cases	Prioritisation of complex cases on agenda to enable sufficient time for their discussion	No attempt is made to order cases in terms of complexity and an inappropriate amount of time is spent on cases (i.e. too much or too little)	Some attempt is made to order cases in terms of complexity but an inappropriate amount of time is spent discussing some of the cases	Patient cases are discussed in a clear order but time is used inappropriately in some cases	Patient cases are discussed in a clear order and an appropriate amount of time is spent discussing each case	
		(1)	(2)	(3)	(4)	
Organisation/admin during meetings:						
a) patient notes	Availability of patient notes	No patient records available at meeting	Some required past/current reports not available	Hardcopy and all necessary past/current reports available	Electronic access to patient notes and all necessary past/current reports available	
		(1)	(2)	(3)	(4)	
b) case presentation	Comprehensiveness and coherence of case presentation	Rambling; entirely reading from notes; does not seem familiar with patient	Some evidence of familiarity with patient and info presented in reasonable fashion		Comprehensive succinct coherent presentation (evidence of familiarity with patient and findings)	
		(1)	(2)		(3)	
**Clinical decision making**						
Patient centred care	Includes mention of patient-based information (e.g. demography; co-morbidities; psycho-social or supportive needs; patient wishes/family preferences)	Patient-centred factors sufficiently acknowledged in less than 20% cases	Patient-centred factors sufficiently acknowledged in less than 50% cases	Patient-centred factors sufficiently acknowledged in 50% + cases (but not all cases)	Patient-centred factors sufficiently acknowledged in all cases	
		(1)	(2)	(3)	(4)	
Treatment plans	Clarity of treatment plan	Treatment plan not discernable	Treatment plan communicated verbally	Treatment plan communicated verbally and recorded	Treatment plan communicated verbally, recorded with a clearly articulated plan regarding the next steps.	
		(1)	(2)	(3)	(4)	
**Characteristic of Effective MDT-working**	**Quality Criteria**	**Quality of team-working (score in brackets)**				
**Teamworking & culture**		**severe and sustained conflict**	**overt conflict un-sustained**	**tension sustained**	**tension un-sustained**	**no tension**
		**(−4)**	**(−3)**	**(−2)**	**(−1)**	**(0)**
d) tension/conflict	Extent of tension and/or conflict observable in the team	≥1clear example of conflict observed which persists throughout meeting	≥1clear example of conflict observed does not persist throughout meeting	≥1 instance of tension observed which persists throughout meeting	≥1 instance of tension observed but does not persist throughout meeting	No tension

Quality criteria for each characteristic were initially informed by relevant literature, observation of MDMs in a range of tumour types, and review of the survey data that had informed the Characteristics [18]. These prototype quality criteria were subsequently reviewed by an independent panel consisting of a consultant radiologist, oncologist and nurse with bowel cancer expertise as well as a senior NHS cancer manager. The panel discussed the potential variation in quality for each characteristic. Optimal ratings were calibrated against the recommendations in the Characteristics. Thresholds for lower quality performance were based on consensual agreement about the added value or detriment to MDT-working of variations to the optimal rating. It was agreed that the variation in quality for most characteristics would best be represented on a four-point scale: very poor (1), poor (2), good (3) or very good (4). Very poor/poor were merged for three characteristics where it was felt that further categorisation would lose value (Table 1).

Video-taped observation was proposed for rating behavioural characteristics such as chairing, teamwork and decision-making, to enable multiple observers to rate identical footage and allow replay. To enhance the reliability of behavioural ratings a case discussion proforma was deve-loped for rating characteristics on a case-by-case basis prior to being aggregated to an overall rating (Additional file 1). Aggregation was a qualitative process for most characteristics: forming global judgments on the rating that best reflected performance based on notes taken for individual cases. The only exception was patient centred care where the rating was based on quantification of objective content. This was because it would be necessary to know the patient in order to value-judge this characteristic of effectiveness. Rating is instead based on the frequency that patient-based information is considered (Table 1). Aspects of team functioning that could not easily be observed on a video-taped recording and could be objectively described were rated on the basis of notes and/or information collected by the researcher at the time of the videotaped meeting. This included attendance, the meeting agenda, meeting venue and technology. The researcher who was present at the meeting verified attendance information from the MDT lead and/or MDT coordinator, obtained an anonymised hard copy of the meeting agenda, and recorded information about the meeting venue and technology on a study-specific proforma (Additional file 2) to ensure systematic recording of the detail required for assessing these criteria.

### Piloting MDT-OARS: acceptability and variation in performance

Ten bowel cancer teams volunteered to participate (ascertained via their participation in another study [20]). One MDM per team was filmed and observed in-vivo. Quality was subsequently assessed using completed case discussion proformas, the completed in-vivo proforma and anonymised agenda (Table 1, Additional files 1 and 2). Short structured interviews were conducted with a range of members from each team (including at minimum the MDT lead, MDT coordinator and clinical nurse specialist) to determine the impact of the observational method on the meeting, and confirm attendance information.

Team members provided written informed consent prior to the meeting being video-recorded and all patient discussions and agendas were anonymised. Ethics approval was granted by the South East Multi-Centre Research Ethics Committee and R & D approval was obtained from the relevant NHS Trust for each MDT.

#### *Analysis*

Quality ratings for each characteristic are presented in their unstandardized form and are summed to provide an overall score out of 58 (Table 2). The ratings for all characteristics except *teamworking: presence of tension/conflict* were standardised to the same scale and either presented on a scale of 1–4 or dichotomised (very poor/poor vs. good/very good) to enable visual comparison of variability in quality within and between teams. Rating of the presence of tension/conflict is not presented graphically as the scale was not compatible.

**Table 2:** Quality of teamworking in 10 bowel cancer teams: unstandardized scores

**Characteristic of effective teamworking**	**Rating scale**	**Team**										**Total number of teams ‘very good’**
		**1**	**2**	**3**	**4**	**5**	**6**	**7**	**8**	**9**	**10**	
		**Score (see Table 1 for criteria)**										
**The Team**												
Attendance	1 to 4	4	4	3	1	2	2	4	4	4	4	**6**
Leadership: chairing of meeting	1 to 4	2	4	1	3	2	2	3	3	3	2	**1**
Teamworking & culture:												
a)Inclusion of relevant team members	1 to 4	2	2	2	4	2	3	3	2	4	4	**3**
b) Team Sociability	1 to 4	1	3	2	4	3	2	3	1	4	2	**2**
c) Mutual respect	1 to 4	2	4	2	4	2	2	3	2	4	4	**4**
d) tension and conflict	0 to −4	0	0	−1	0	0	−1	0	−1	0	−1	**6**
Personal development & training	1 to 3	1	3	2	2	2	1	1	2	2	2	**1**
**Infrastructure for meetings**												
Meeting venue	1 to 4	2	3	3	1	2	3	4	3	4	3	**2**
Technology & equipment	2 to 9	7	9	5	3	3	7	7	6	9	7	**2**
**Meeting organisation and logistics**												
Preparation prior to meetings:												
a) agenda	1 to 3	3	3	2	3	2	3	3	3	3	2	**7**
b) Prioritisation of complex cases	1 to 4	1	2	1	1	2	2	2	2	2	1	**0**
Organisation/admin in MDM a) patient notes	1 to 4	3	3	2	2	2	3	3	3	3	3	**0**
b) Case presentation	1 to 3	2	3	1	3	2	2	2	2	3	2	**3**
**Clinical decision making**												
Patient-centred care	1 to 4	1	3	2	2	3	2	2	1	2	2	**0**
Treatment plans	1 to 4	2	4	2	4	1	3	1	2	3	3	**2**
**Total rating**	**11-58**	**33**	**50**	**29**	**37**	**30**	**36**	**41**	**35**	**50**	**40**	

### Feasibility and inter-rater reliability

Feasibility was estimated by collecting data on the time taken to complete observational assessments. Inter-rater reliability was assessed by comparing the ratings of the research team with those of two independent observers: one oncology specialist registrar and one post-doctoral research psychologist. Intraclass correlation coefficients were calculated (two-way mixed models with measures of absolute agreement). 95% confidence intervals are shown. A significance level of p = 0.05 was used. Data were analysed using SPSS v.15.0 for windows.

## Results

### Criteria for assessing MDM quality

The quality criteria and methods for assessing each characteristic are summarized in Table 1.

### Acceptability and team meeting characteristics

Presence of an observer and videocamera in MDT meetings was acceptable to team members and feasible to implement: it caused no delay or interruption to the usual flow of meetings. Interviews with team members confirmed all meetings had been typical: the presence of the researcher and camera had negligible impact on team member behaviour.

Meetings ranged in size from having 10 to 45 core and extended members present (median 16 members). The largest MDT meeting was a network-wide meeting comprising three local MDTs; all others comprised one local MDT. The average meeting length was 53minutes (range: 25–86 minutes) and teams discussed an average of 13 patients (range: 6–21 patients). Each patient was discussed for an average of 4.5 minutes (range 3–9 minutes).

### Variation in team performance between teams

There was wide diversity in ratings between teams across all characteristics. Total scores (out of a possible 59) ranged from 29 (team 3) to 50 (teams 2 and 9; Table 2). Only one team (Team 3) failed to achieve ‘very good’ criteria for at least one characteristic. The quality of teamwork in this team was particularly poor in relation to chairing (the chair allowed long pauses in discussions and did not explicitly confirm treatment plans before moving onto the next case); prioritisation of the agenda (9/14 cases discussed were rated as having too much time spent on them as key information or results were missing, or because relevant team members, for example the oncologist, were absent); and presentation of clinical information (case presentations were unprepared and unstructured, time was spent searching for relevant information for most cases). In contrast, Teams 2 and 9 met ‘very good’ criteria for 9 out of the 15 characteristics. These teams demonstrated: optimal chairing (by ensuring discussions remained focused and clearly articulating treatment plans); inclusion of all relevant team members in discussions (most other teams, although not always dominated by one or two members, particularly lacked contribution from the clinical nurse specialist or any other nurses); explicit use of the meeting for professional development (a well prepared, informative discussion about a waiting times breach resulting in clear actions); optimal venue and technology (a spacious purpose-built room; core team seated in horseshoe formation enabling sight of imaging/pathology and each other. Picture Archiving and Communications System (PACS) and multiple screens facilitated viewing of current and retrospective pathology and radiological imaging); circulation of a comprehensive agenda prior to meetings (including patient ID and demographics, all previous relevant history, tests undertaken/results, reason for MDT discussion, and space for MDT recommendation to be inserted).

### Variation in team performance within teams

**Figure 1:**
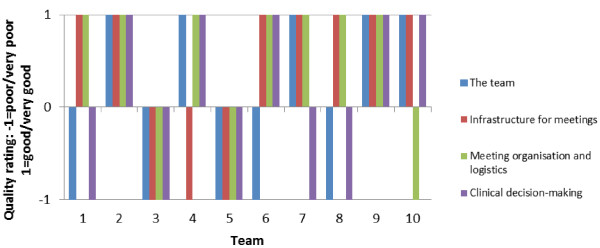
Quality of teamworking across the four domains of teamworking.

Some internal consistency within teams was evident: MDTs performing well in one domain tended to perform well in other domains (and vice versa, Figure 1). Eight out of the 10 teams had the same rating (either ‘very good/good’ or ‘very poor/poor’) for at least three of the four domains of teamworking. The internal consistency was most evident in teams performing at either end of the spectrum: Team 3 received consistently poor ratings across all characteristics with three ‘very poor’ ratings and no ‘very good’ ratings, and teams 2 and 9 received 9 ‘very good’ ratings and had no ‘very poor’ ratings of quality for any characteristic (Table 2).

### Characteristics of effectiveness most and least likely to be achieved

**Figure 2:**
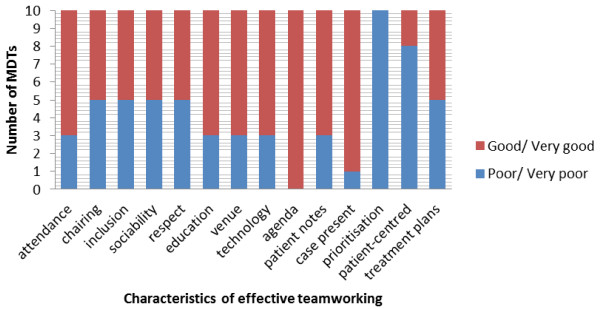
Variation in quality of team performance according to characteristic of effective teamworking.

Teams were most likely to achieve ‘very good’ ratings for: administrative preparation (a comprehensive agenda), membership/attendance (all core team members or deputies present for whole meeting), and tension/conflict (no tension or conflict in meetings) (Table 2, Figure 2). Teams were least likely to achieve ‘very good’ ratings for: patient-centredness of case discussions and prioritisation of cases on the agenda. No teams were observed to explicitly consider patient-centred factors in all patient discussions: most teams considered patient-based factors in less than half of cases, mostly comprising demographic information rather than holistic needs or preferences. There was no attempt by any team to use the agenda to prioritise the order of case discussions to ensure appropriate time was spent discussing complex cases. In all teams at least some cases were judged as being discussed for too little or too much time based on their complexity.

### Feasibility and inter-rater reliability

Completion of assessments required 93 minutes on average (range: 45–160 minutes). There was no consistent difference in time taken by different observers; instead the time taken was associated with meeting length, averaging between 1.25 and 2 times the meeting length. There was a trend for assessments to take longer initially (average 2 times length of meeting) compared to the final few teams observed (average 1.25 times length of meeting).

Acceptable intra-class correlation coefficients of at least 0.50 (and up to 0.92) were achieved for all except two characteristics of effective teamworking: the absence of tension/conflict (ICC = 0.10) and evidence of treatment planning (ICC = 0.32; Table 3).

**Table 3:** Inter-rater reliability of ratings across three independent observers

**Rating Scale domains**	**Intraclass correlation coefficient (95% confidence interval)**	**Significance (p)**
**THE TEAM**		
Attendance	*n/a – rating based on in-vivo factual information*	
Leadership: chairing of the meeting	0.58 (−0.37 to 0.90)	0.07
Teamworking & culture:		
a) Inclusion of all relevant team members	0.68 (0.07 to 0.92)	0.02
b) Team sociability	0.52 (−0.56 to 0.89)	0.10
c) Mutual respect between participants	0.72 (0.21 to 0.93)	0.01
d) Tension/conflict	0.10 (−0.92 to 0.73)	0.40
Personal development and training	0.74 (0.23 to 0.93)	0.01
**INFRASTRUCTURE FOR MEETINGS**		
Meeting venue	0.92 (0.76 to 0.98)	<0.001
Technology & equipment	0.92 (0.76 to 0.98)	<0.001
**MEETING ORGANISATION AND LOGISTICS**		
Preparation prior to meetings: a) agenda	0.55 (−0.13 to 0.87)	0.04
b) prioritisation of complex cases	*n/a rated by one observer only*	
Organisation/admin during meetings: a) patient notes	0.47 (−0.27 to 0.85)	0.07
b) case presentation	0.74 (0.16 to 0.94)	0.01
**CLINICAL DECISION-MAKING PROCESS**		
Patient-centred care	0.77 (0.24 to 0.95)	0.01
Treatment Plan	0.32 (−0.84 to 0.83)	0.23

## Discussion

We have developed a novel, observational measure for assessing quality in cancer MDT meetings. The quality criteria are calibrated against ‘effectiveness’ as defined by consensus from over 2000 MDT members, and measure quality in relation to 15 aspects of observable activity in MDT meetings. Our preliminary study has shown that independent observation of MDT meetings is acceptable to teams and feasible to conduct. Whilst only a small number of teams participated in this pilot study, the application of the measure highlighted wide diversity in the quality of teamworking across the range of characteristics measured.

Teams typically performed well in terms of the administrative preparation for meetings and having the appropriate team members in attendance. They performed less well in relation to spending adequate time discussing cases (requiring case prioritisation on the agenda), and having patient-centred case discussions. Both of these aspects of MDT function require additional preparation time, either in relation to compiling and assessing case information to determine their complexity, and/or additional time with patients to comprehensively assess their history, needs and wishes. The importance of undertaking regular assessment of patients' needs and preferences (now referred to as holistic needs assessment [21]) was highlighted in the NICE guidance for improving supportive and palliative care [22] and generally it is expected Clinical Nurse Specialists (CNS) should lead this. Ensuring patient-led decision making is fostered in the MDT meetings, where patients are not present, is challenging due to the time pressures of meetings and requires further attention. Nevertheless if the opportunity is not grasped for information to be obtained and shared with the wider team at the earliest opportunity it may fail to impact on decision making, or may cause avoidable delays to treatment [9,10]. In this study, few nurses contributed to case discussions which may at least in part explain the lack of patient-based information discussed. Similar findings have been reported elsewhere [23]. Although responsibility for ensuring that recommendations are patient-centred rests with the whole team, training and support to enhance nurses’ involvement in MDT discussions may be warranted.

This pilot study was aimed at determining ‘proof of concept’ and has demonstrated that it is acceptable and feasible to measure complex aspects of team behaviour and activities such as leadership, teamworking and decision-making. The calibration of quality criteria against characteristics of effectiveness agreed by a large sample of MDT members, in addition to using available evidence and expert input, ensured content validity. Furthermore, most characteristics were measured reliably in the hands of different observers. There was low agreement in ratings for the presence of tension/conflict and the clarity of treatment recommendations which may be related to level of clinical experience. The quality criteria require refinement to increase their reliability or it may be necessary for observers to have relevant clinical experience for the ratings of these aspects of teamworking to be valid.

Further testing is required with more teams and other tumour types. Only one meeting was assessed per team which may not have adequately represented their teamworking. Team members confirmed that the filmed meetings were typical of their usual meetings in all cases, but some aspects of teamworking may have been more reliably rated longitudinally. This may be particularly important where behaviour or performance receives a poor rating. Furthermore, the teams volunteered through their participation in another study. It is necessary to test this method of observational assessment with other teams to further confirm its acceptability. Validation of ratings against other subjective and objective outcomes (such as team member assessments of their own performance, clinical outcomes, peer review data & national patient experience data) will be important to further define the characteristics of effective teamworking. The current design of the tool, based upon rating case-by-case, enhanced objectivity of ratings but was time consuming and is likely to require simplification to have clinical as well as research utility. Together with further validation, it may be desirable to develop quality criteria for other characteristics of MDM effectiveness. In particular this could include other aspects of case discussions such as presentation of nationally agreed minimum datasets for radiology, pathology and clinical data; and adherence to relevant nationally and locally agreed protocols. Such assessment may require observers with clinical expertise, at least for assessment of these aspects of team functioning.

The variation in quality of teamworking we report reinforces the need to provide teams with appropriate assessment tools, resources and training to optimise their performance. Indeed 85% of MDT members that responded to the UK national survey agreed MDTs need performance measures [18].

## **Conclusion**

The preliminary testing of MDT-OARS indicates this could be a useful component of MDT assessment, alongside other measures of the quality of patient care provided by MDTs including peer review and patient experience surveys.

## Misc

This study was funded by Cancer Research UK and by the COMPASS Collaborative, a National Cancer Research Institute Supportive and Palliative Care Research Collaborative.

## Competing interest

The authors declare they have no competing interests.

## Authors' contributions

All named authors have agreed to the submission of this manuscript and participated in this study to a sufficient extent to be named as authors. AJR, AR and CT conceived the idea for the study; CT was the study lead and wrote the first draft of the paper. All authors participated in the development and/or application of the quality criteria, the critical revision of the article and have read and approved the final manuscript.

## Role of funding source

The funding bodies had no role in the study design; collection, analysis, interpretation of data; writing the report; or the decision to submit for publication**.**

## Pre-publication history

The pre-publication history for this paper can be accessed here:

http://www.biomedcentral.com/1471-2407/12/202/prepub

## Supplementary Material

Additional file 1 Measuring the quality of MDT working: an observational approach (Taylor et al, BMC Cancer). Click here for file

Additional file 2 Case discussion proforma *(to be completed for each case discussion observed)*. Click here for file
