# Supplementary material for: Measuring the quality of MDT working: an observational approach
Source: BMC Cancer. 2012 May 29;12:202. doi: 10.1186/1471-2407-12-202 (PMC3489862; doi:10.1186/1471-2407-12-202)
Supplement: Additional file 1 — Measuring the quality of MDT working: an observational approach (Taylor et al, BMC Cancer). [file 1471-2407-12-202-S1.doc]

**MDT-OARS**

**(Observational Assessment Rating Scale)**

**In-vivo proforma**

**Team Information**

**Team ID number: Date of observation:**

| **Team composition details** | |
| --- | --- |
| **Total number of people present at the meeting** |  |
| **Are any core members missing?**  (Check meeting attendance list/confirm with MDT co-ordinator to assess this. See below for guidance on who constitutes core member) |  |

| **Core members of colorectal MDT (taken from Improving Outcomes Guidance for Colorectal Cancer)** |
| --- |
| **At least two specialist surgeons**  **Oncologist**  **Diagnostic radiologist with gastro-intestinal expertise**  **Histopathologist**  **Skilled colonoscopist of any discipline (surgeon, physician or specialist nurse)**  **Clinical Nurse Specialist (CNS) – *focus on providing information and support***  **Palliative care specialist (doctor or nurse) who should work with palliative care services in the community**  **Meeting co-ordinator**  **Team secretary [in small teams the MDT co-ordinator may act as secretary]** |

**Layout of Room**

Please sketch a layout of the room in the box below in regards to positioning of table(s), layout of seating, and screens and identify where core members are seated.

**Code:**

LC: lead clinician (specialty in brackets e.g. if a surgeon LC (S)

S: Surgeon R: Radiologist

G: Gastroenterologist P: Pathologist

CNS: Clinical Nurse Specialist O: Oncologist

N: other nurse

MDT: MDT coordinator

NB: if LC is not the Chair please indicate who the chair is.

**SUPPORTING INFRASTRUCTURE**

| **Meeting venue:**  Describe room size and layout if not obvious from layout of room overleaf  Describe lighting, location, temperature, ability to view diagnostics |
| --- |
|  |

| **Technology and equipment:**  Describe imaging facilities (PACS? Lightbox? Current and retrospective imaging?)  Describe pathology facilities (Microscope? Projection of slides? Current and retrospective?  Availability of multiple screens for simultaneous viewing? Y/N |
| --- |
|  |
