# Supplementary material for: Measuring the quality of MDT working: an observational approach
Source: BMC Cancer. 2012 May 29;12:202. doi: 10.1186/1471-2407-12-202 (PMC3489862; doi:10.1186/1471-2407-12-202)
Supplement: Additional file 2 — Case discussion proforma (to be completed for each case discussion observed). [file 1471-2407-12-202-S2.doc]

# Additional File 2: Case discussion proforma *(to be completed for each case discussion observed)*

**Team: Start/ finish time:**

**Patient no: Total time:**

**Number of patients on agenda:**

**Number of unscheduled patients:**

**Patient status:** (1) pre-treatment; (2) post-treatment; (3) recurrence; (4) advanced disease

**Reason for bringing to MDM (describe/unknown):**

| **Case discussion** |  | |
| --- | --- | --- |
|  | Team members involved | **Detail** |
| **Chairing** *(introduces, asks for input, summarizes)* |  |  |
| **Case presentation** *(e.g. preparation; info easily found and given coherently and comprehensively; evidence of familiarity with patient)* |  |  |
| Presentation of diagnosis/staging *(inc whether path/rad reports are presented/missing etc)* |  |  |
| Treatment planning/decision-making (inclusiveness; consensual decision) |  |  |
| **Content of discussion** | **Please tick** | |
| **Psychosocial issues, e.g. family support, mental health concerns** | **🞎** | |
| **Demography, e.g. age** | **🞎** | |
| **Co-morbidities** | **🞎** | |
| **Patient wishes/ family preferences** | **🞎** | |
| **Discussions of research, e.g. enrolling patient in a trial or research evidence** | **🞎** | |
| **Team-working** |  |  |
| **Discussion/challenge of previous diagnostic decisions** | Y/N Who? |  |
| **Disrespect (e.g concurrent discussion, interruption, not valuing contributions)** | Y/N Who? |  |
| **Tension & conflict** | Y/N  Who between? |  |
| **Team sociability** | Y/N: Who? |  |

**Final management recommendation (describe including how it is summarized and who by):**

**Clear? Y/N Agreed by All? Y/N**

**Complexity of case:** complex/routine/other **Time spent discussing case:** too much/too little/about right
